# Long Non-coding RNA SNHG17 Promotes Cell Proliferation and Invasion in Castration-Resistant Prostate Cancer by Targeting the miR-144/CD51 Axis

**DOI:** 10.3389/fgene.2020.00274

**Published:** 2020-04-15

**Authors:** Minghua Bai, Yutiantian Lei, Mincong Wang, Jinlu Ma, Pengtao Yang, Xingyi Mou, Yiping Dong, Suxia Han

**Affiliations:** ^1^Department of Radiation Oncology, The First Affiliated Hospital of Xi’an Jiaotong University, Xi’an, China; ^2^Department of Radiation Oncology, The Second Affiliated Hospital of Xi’an Jiaotong University, Xi’an, China; ^3^Department of Clinical Medicine, Xi’an Jiaotong University Health Science Center, Xi’an, China

**Keywords:** SNHG17, CD51, miR-144, CRPC, proliferation

## Abstract

Previously, we found that the expression of long non-coding RNA (lncRNA) small nucleolar RNA host gene 17 (SNHG17) was up-regulated in castration-resistant prostate cancer (CRPC) cells compared to that in hormone sensitive prostate cancer (HSPC) cells. Moreover, we found that CD51 was up-regulated in prostate cancer cells and promoted the carcinogenesis and progression of prostate cancer. However, the regulatory mechanism of SNHG17 and CD51 in the development of CRPC remains unclear. In the current study, we aimed to elucidate the expressions, functions, and underlying mechanism of SNHG17 and CD51 in CRPC. Our results further confirmed that both SNHG17 and CD51 were up-regulated in CRPC tissues and cells. In addition, we found that SNHG17 expression was positively correlated with CD51 expression in prostate cancer. Mechanically, SNHG17 functioned as a competing endogenous RNA (ceRNA) to up-regulate CD51 expression through competitively sponging microRNA-144 (miR-144), and CD51 was identified as a direct downstream target of miR-144 in CRPC. Functionally, down-regulation of SNHG17 or up-regulation of miR-144 inhibited the proliferation, migration, and invasion of CRPC cells, whereas up-regulation of SNHG17 and down-regulation of miR-144 promoted the proliferation, migration and invasion of CRPC cells *in vitro* and *in vivo*. Using gain and loss-of function assay and rescue assay, we showed that miR-144 inhibited cell proliferation, migration and invasion by directly inhibiting CD51 expression, and SNHG17 promoted cell proliferation, migration and invasion by directly enhancing CD51 expression in CRPC cells. Taken together, our study reveals the role of the SNHG17/miR-144/CD51 axis in accelerating CRPC cell proliferation and invasion, and suggests that SNHG17 may serve as a novel therapeutic target for CRPC.

## Introduction

It is estimated that there will be 174,650 newly diagnosed prostate cancer cases and 31,620 estimated prostate cancer related deaths in 2019 in the United States, accompanied by drastically increased incidence and mortality in China in the last decade (Chen et al., 2017; Siegel et al., 2019). Despite continuous improvements in diagnosis and treatment, the recommended maintenance schedules from androgen deprivation therapy (ADT) to radical resection are at present only effective in patients with hormone-sensitive prostate cancer (HSPC) (Rice et al., 2019). This is when the tumor has progressed to castration-resistant prostate cancer (CRPC) stage, and the 5 years overall survival rate remains extremely disappointed (Nuhn et al., 2019). Hence, exploring new detailed mechanisms that account for CRPC progression is of great importance.

Numerous evidences have confirmed that long non-coding RNAs (lncRNA) played an important role in CRPC by functioning as oncogenes or tumor suppressors. For example, lncRNA LBCS was shown to suppress castration resistance and proliferation of prostate cancer cells by functioning as a scaffold of hnRNPK (Heterogeneous nuclear ribonucleoprotein K) protein and AR (Androgen receptor) mRNA to inhibit AR translation efficiency (Gu et al., 2019). Moreover, lncRNA small nucleolar RNA host gene 15 (SNHG15) has been shown to play oncogenic roles in the progression of prostate cancer (Zhang Y. et al., 2019). It was shown that SNHG15 served as a competing endogenous RNA (ceRNA) to promote the proliferation, migration, and invasion of prostate cancer cells by targeting microRNA(miR)-338-3p/FKBP prolyl isomerase 1A (Zhang Y. et al., 2019). Previously, we showed that the expression of long intergenic non-coding RNA 00963 (Linc00963) was abnormally up-regulated in CRPC cells by generating differentially expressed lncRNA profiles of LNCaP and C4-2 cells (Wang et al., 2014). Furthermore, we also found that the expression of lncRNA small nucleolar RNA host gene 17 (SNHG17) was up-regulated in C4-2 cells compared to that in PC-3 cells (Wang et al., 2014), thus indicating that SNHG17 may be involved in the transition from HSPC to CRPC. However, the expressions SNHG17 in CRPC tissues and its function in CRPC progression have not been fully investigated.

CD51, also known as integrin subunit alpha V (ITGAV), is a member of the integrin alpha chain family (Conroy et al., 2016). Several evidences have indicated that the up-regulation of CD51 is significantly associated with cancer progression and poor prognosis in multiple types of cancers, such as gastric cancer (Wang et al., 2019), colorectal cancer (Flum et al., 2018), laryngeal carcinoma (Lu et al., 2009), and hepatocellular carcinoma (Kang et al., 2019). Mechanically, it was confirmed that CD51 could inhibit the expression of *E*-cadherin and promote epithelial-to-mesenchymal transition (EMT) by interacting with transforming growth factor-beta (Feldkoren et al., 2017). Further, it was found that treatment with CD51 antagonist could suppress *de novo* formation and progression of bone metastases in CRPC by inhibiting EMT process and decreasing the prostate cancer stem cell population (pCSC) population (van der Horst et al., 2011). Interestingly, treatment with a humanized CD51 monoclonal antibody also showed excellent clinical benefit in some CRPC patients with bone metastases in a multicenter phase I&II study (Wirth et al., 2014; Hussain et al., 2016). We also found CD51, which was down-regulated by p53 at transcriptional levels, was required for prostate cancer stemness and could enhance cancer initiation, metastatic potential, and chemoresistance (Sui et al., 2018). However, the regulation of CD51 in CRPC cells at the post-transcriptional levels remains unclear.

In the current study, we showed that SNHG17 and miR-144 could regulate CD51 expression at post-transcriptional levels by functioning as ceRNA. Besides, CD51 was identified as the downstream effector and functional mediator of SNHG17 and miR-144 in CRPC. In addition, we found that SNHG17 promoted CRPC cell proliferation, migration and invasion *in vitro* and *in vivo* by targeting miR-144/CD51 axis. Hence, our study revealed the role of the SNHG17/miR-144/CD51 axis in accelerating CRPC cell proliferation and invasion, and suggested that SNHG17 may serve as a novel therapeutic target for CRPC.

## Materials and Methods

### Human Patient Samples

Samples of 46 patients with CRPC and 149 patients with HSPC were provided by The First Affiliated Hospital of Xi’an Jiaotong University. The clinical-pathological features of prostate cancer patients enrolled in this study were described in our previous study (Sui et al., 2018).

### Cell Culture

Human prostate cancer cell lines LNCaP, C4-2, PC-3, and DU145 were purchased from GeneChem (Shanghai, China). LNCaP, DU145, C4-2 and PC-3 cells were cultured in Dulbecco’s modified eagle medium (DMEM, Gibco) containing 10% fetal bovine serum (FBS, Cellmax, Beijing, China), 1% penicillin-streptomycin (Cellmax) at 37°C in a humidified atmosphere of 5% CO_2_.

### Construction of Lentivirus Expression Vector

Lentiviral-SNHG17 (Lv-SNHG17), Lentiviral-CD51(Lv-CD51), and lentiviral scrambled negative control (Lv-control) were designed and provided by Genechem (Shanghai, China). Briefly, the full length of human SNHG17 (transcript variant 2^1^), CD51 and scramble control were cloned intro Bam I and *Age*I sites of CV146 core vector (Ubi-MCS-SV40-Firefly-Luciferase-IRES-Puromycin). Then, 20 μg CV146-SNHG17/CD51/control, and lentiviral packaging helper plasmid pHelper 1.0 (15 μg) and pHelper 2.0 (10 μg) were co-transfected into 293T cells by Lipofectamine 2000. The medium changed to 10% DMEM 8 h later and the cell supernatant was collected 72 h later, then centrifugation happened at 4°C for concentration and purification of Lv-SNHG17, Lv-CD51, and Lv-control.

### Lentivirus Infection and siRNA/miRNA Transfection

The lentivirus infection and siRNA/miRNA transfection were performed as our previous study described (Sui et al., 2018; Mu et al., 2019). Briefly, for lentivirus infection, PC-3 or C4-2 cells were seeded in six-well plates and infected by HiTransG A (Genechem) according to the manufacturer’s protocol. Then, PC-3 and C4-2 cells were selected with puromycin for 2 weeks to remove uninfected cells and obtain stable SNHG17 up-regulated cells. The stable SNHG17 up-regulated cells were collected for WB, RT-QPCR, EdU, migration and invasion assay. CD51 siRNA, scrambled NC siRNA, miR-144 mimics, miR-144 inhibitors, and miR-144 NC were synthesized and provided by Ribo Bio (Guangzhou, China). For siRNA/miRNA transfection, PC-3 and C4-2 cells were seeded in six-well plates and transfected by Lipofectamine 2000 (Thermo Fisher Scientific, United States) according to the manufacturer’s protocol. Cells were collected for RT-QPCR, WB, EdU assay, wound healing assay, and transwell assay 48 h later. The sequences for lentivirus and siRNAs used in our study are provided in Table 1.

**TABLE 1:** Sequence of lentivirus and siRNAs used in the experiments.

Name	Sequence
Lv-SNHG17 sense	5′-AGGTGA-CCGGTGCCATGTTGATTGTCGCTTTTG-3′
Lv-SNHG17 antisense	5′-CACAG-GATCCAGCCTAAGTCCGAAGACTCGAT-3′
Lv-CD51 sense	5′-AGGTGA-CCGGTCCGTGTCACGAGATACCTTTAA-3′
Lv-CD51 antisense	5′-CACAG-GATCCGTCTTACGCTACCCTGTGAGAGAT-3′
Lv-control sense	5′-AGGGTACCCCTGGGACCCGGCACCGGAGACG-3′
Lv-control antisense	5′-TCCACTAGGTGCGGACCGCTCCCAATGAG CA-3′
SNHG17 siRNA-1 sense	5′-GAUUGUCAGCUGACCUCUGUCCUGU-3′
SNHG17 siRNA-1 antisense	5′-ACAGGACAGAGGUCAGCUGACAAUC-3′
SNHG17 siRNA-2 sense	5′-AUCCUCAUGUCACUGUCUUGUUCCTT-3′
SNHG17 siRNA-2 antisense	5′-UGAACGCCGAGCUGCGCCUUAAGUUA-3′
CD51 siRNA sense	5′-CCAACUUCAUUAUAGAUUUTT-3′
CD51 siRNA anti sense	5′-AAAUCUAUAAUGAAGUUGGTA-3′
NC siRNA sense	5′-UUCUCCGAACGUGUCAGGUTT-3′
NC siRNA antisense	5′-AGGUGACACGUUCGGAGAATT-3′
miR-144 mimics	5′-UACAGUAUAGAUGAUGUACU-3′
miR-144 inhibitors	5′-AGUACAUCAUCUAUACUGUA-3′
miR-144 NC	5′-UUUGUACUACACAAAAGUACUG-3′

### EdU Staining

Cell proliferation of different transfected PC-3 and C4-2 cells was evaluated using Cell-Light EdU Apollo567 *in vitro* Kit (Ribo Bio) according to the manufacturer’s instructions. 10^5^ cells were seeded in 96-well plates and stained with 100 μL 50 μM EdU solution for 2 h in the dark at room temperature. Then, the cells were fixed with 4% paraformaldehyde for 30 min and permeabilized with 0.5% Triton X-100 for 15 min. After washing three times with PBS, the cells were stained with Apollo^®^567 and DAPI. Representative images were taken using the confocal microscope (Olympus, Japan) at ×200 magnification.

### Wound Healing Assay, CCK-8, Transwell Assay, and Western Blot (WB)

Cell proliferation of different transfected PC-3 and C4-2 cells was further evaluated using CCK-8 assay. The migrative abilities of different transfected PC-3 and C4-2 cells were measured by wound healing assay. The invasive abilities of different transfected groups were measured by transwell assay. Protein levels of CD51 in different transfected PC-3 and C4-2 cells were measured by WB. All the procedures for wound healing, transwell assay, and WB were performed as our previous study described (Sui et al., 2018).

### RNA Pull-Down Assay

RNA pull-down assay were performed as our previous study described with a few modifications (Mu et al., 2019). Briefly, PC-3 cells were lysed in NP40 lysis buffer, and 1 mg cell extracts were incubated with biotin-labeled SNHG17-probe or SNHG17-MUT-probe at 4°C for 6 h. Subsequently, the RNAs with biotin-labeled NC (Bio-NC-probe), SNHG17 (Bio-SNHG17-probe), or SNHG17-MUT (Bio-SNHG17-MUT-probe) were mixed with 40 μl streptavidin agarose beads and incubated on a rotator overnight. At last, the expression of miR-144 in the retrieved RNA was identified using RT-QPCR.

### Luciferase

Luciferase assay was performed as our previous study described with a few modifications (Mu et al., 2019). Briefly, PC-3 cells were seeded in a 96-well plate at 70% confluence. The sequence of SNHG17 and 3′-untranslated region (UTR) of CD51 containing miR-144-binding sites were cloned into pMirGLO dual-luciferase miRNA target expression vector (Promega Corporation, WI, United States), yielding pmirGLO-wild type (WT)-SNHG17/CD51. Mutations from U to A were introduced into potential miR-144 binding sites of SNHG17, and mutations from U to C were introduced into potential miR-144 binding sites of CD51 using a QuikChange^TM^ site-directed mutagenesis kit (Stratagene; now owned by Agilent Technologies, Inc., Santa Clara, CA, United States) to construct pmirGLO-mutant (MUT)-SNHG17/CD51. Subsequently, 20 nM miR-144 NC/mimics and 50 ng pmirGLO-WT/MUT-SNHG17/CD51 were co-transfected into PC-3 cells using Lipofectamine 2000. Cells were collected 48 h after transfection, and the relative firefly luciferase activities were measured using a dual-luciferase reporter assay system according to the manufacturer’s protocol (Promega Corporation). *Renilla* luciferase activity served as an internal control.

### Anti-Ago2-RIP

The RIP assay were performed as our previous study described with a few modifications using the EZ-Magna RIP kit (Merck Millipore, MA, United States) following the manufacturer’s protocols (Miao et al., 2019). PC-3 cells were transfected with SNHG17 siRNA or SNHG17 NC; C4-2 cells were infected with Lv-SNHG17 or Lv-control. Forty eight hour later, cells were lysed in RIP lysis buffer, and cell extracts were incubated with anti-Ago 2 (Merck Millipore) or IgG (Merck Millipore) for 6 h. Then, purified RNA was analyzed by qRT-PCR to identify the presence of SNHG17 and CD51.

### FISH

Oligonucleotide probes for SNHG17 and U6 were purchased for Ribo Bio (Guangzhou, China). The PC-3 cells were seeded in 20 mm confocal dishes. After overnight incubation, the cells were fixed with 4% paraformaldehyde for 20 min and permeated with Triton X-100 for 90 s. Then, PC-3 cells were incubated with hybridization buffer supplemented with CY-3-labeled- SNHG17 and U6 FISH probe at 37°C overnight in a dark moist chamber. The next day, cells were washed three times in 2X SCC and stained with DAPI. The images were acquired using a confocal microscope (Olympus) at ×400 magnification.

### RNA Extraction and Quantitative Real-Time Polymerase Chain Reaction (RT-QPCR)

Cytoplasmic and nuclear RNA were isolated with a Cytoplasmic & Nuclear RNA Purification Kit (Norgen Biotek, ON, Canada), according to the manufacturer’s instructions. Briefly: lying the cells with Lysis Solution for 30 min at room temperature. Then, separating cytoplasmic and nuclear RNA by centrifugation at 13000 *g* for 15 min. Adding the supernatant which contains cytoplasmic RNA to the Bind 1 column, and adding the pellet which contains nuclear RNA to the Bind 2 column to further remove contaminating RNAs. After washing with Wash buffer, eluting the purified cytoplasmic and nuclear RNA with Elution buffer, respectively. Total RNA extraction and RT-QPCR were performed as our previous study described (Sui et al., 2018). The primers used in this study were purchased from Sangon Biotech (Shanghai, China) and displayed in Table 2.

**TABLE 2:** Primers used in the experiments.

Name	Sequence
SNHG17 forward (human)	5′-TGCTTGTAAGGCAGGGTCTC-3′
SNHG17 reverse (mouse)	5′-ACAGCCACTGAAAGCATGTG-3′
β-actin forward	5′-GGCGGCACCACCATGTACCCT-3′
β-actin reverse	5′-AGGGGCCGGACTCGTCATACT-3′
CD51 forward	5′-AAGCTGAGCTCATCGTTTCC-3′
CD51 reverse	5′-GCACAGGAAAGTCTTGCTAAGG-3′
miR-144 forward	5′-GGCCCTGGCTGGGATATCAT-3′
miR-144 reverse	5′-GGTGCCCGGACTAGTACATC-3′
U6 forward	5′-CTCGCTTCGGCAGCACA-3′
U6 reverse	5′-AACGCTTCACGAATTTGCGT-3′

### Animal Experiments

All animal experimental procedures were performed according to the Guidelines for Animal Care and Use of Xi’an Jiaotong University. Each group consisted of 5 BALB/c athymic nude mice (nu/nu; weight: 16-18 g; age: 6 weeks old). In order to investigate the effects of SNHG17 on tumor growth, C4-2 cells with stable upregulation of SNHG17 (5 × 10^6^) or control cells were subcutaneously injected into the left flank of each mouse. After the tumors were visible, the tumor length and width were measured every 3 days for 2 weeks. Tumor volumes were calculated using the formula: 0.5 × length × width^2^. Two weeks later, the mice were sacrificed and the tumor were collected and weighted. The tumor sections were stained with Ki-67 antibody after deparaffinization and rehydration. In order to determine the effects of SNHG17 on cancer metastasis, C4-2 cells with stable upregulation of SNHG17 (5 × 10^5^) or control cells were injected into the tail vein of each mouse. After 3 weeks, or when the mice show a poor overall health condition, the mice were sacrificed and the lungs were collected for further histopathological analysis.

### Statistical Analysis

IBM SPSS statistical software (version 22.0) was used to perform statistical analysis. Student’s *t*-test was used for data analysis of two groups and one-way ANOVA, followed by a least significant difference test which was used for data analysis of multiple groups. *P*-values were determined using 2-sided tests. *P* < 0.05 were considered to have statistical significance.

## Results

### SNHG17 Is Up-Regulated in CRPC and Positively Correlated With CD51 Expression

**FIGURE 1:**
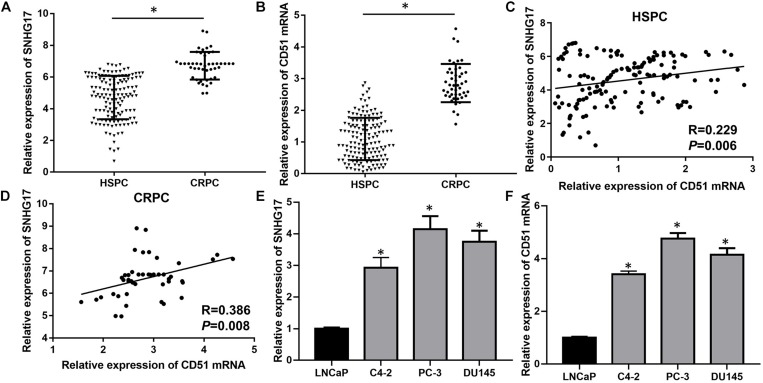
SNHG17 expression was up-regulated and positively correlated with CD51 expression in CRPC tissues and cells. **(A)** The expression of SNHG17 in HSPC and CRPC tissues, as detected by RT-QPCR; **(B)** The mRNA expression of CD51 HSPC and CRPC tissues, as detected by RT-QPCR; **(C)** The association between CD51 and SNHG17 expression in HSPC tissues; **(D)** The association between CD51 and SNHG17 expression in CRPC tissues; **(E)** SNHG17 expression in HSPC cell line (LNCaP) and CRPC cell lines (PC-3 and C4-2), as detected by RT-QPCR; **(F)** The expression of CD51 in HSPC cell line (LNCaP) and CRPC cell lines (PC-3 and C4-2), as detected by RT-QPCR; Data expressed as the mean ± *SE*. *P < 0.05, ns, not significant. The representative results of 3 independent experiments are shown.

Previously, we found that the expression of SNHG17 was up-regulated in the CRPC cell line C4-2 compared to HSPC cell line LNCaP (Wang et al., 2014). Moreover, we found that CD51 was up-regulated in prostate cancer and promoted carcinogenesis and progression of prostate cancer (Sui et al., 2018). Here, we detected the expressions and correlations of SNHG17 and CD51 in CRPC. RT-QPCR analysis revealed that the expression of both SNHG17 and CD51 was up-regulated in CRPC tissues compared to that in HSPC tissues (*P* < 0.05, the expressions of SNHG17 in CRPC and HSPC tissues are shown in Figure 1A, the expressions of CD51 mRNA in CRPC and HSPC tissues are shown in Figure 1B). In addition, correlation analysis indicated a significantly positive correlation between SNHG17 and CD51 mRNA expression in HSPC (*P* < 0.05, Figure 1C) and CRPC cells (*P* < 0.05, Figure 1D). Thus, we further elucidated the regulatory mechanism of SNHG17 and CD51 in CRPC. RT-QPCR analysis revealed that the expression of both SNHG17 and CD51 was up-regulated in CRPC cells (C4-2, PC-3, and DU145) compared to that in HSPC cells (LNCaP) (*P* < 0.05, the expressions of SNHG17 in CRPC and HSPC cells are shown in Figure 1E, the expressions of CD51 mRNA in CRPC and HSPC cells are shown in Figure 1F).

### SNHG17 Promotes Cell Proliferation and Invasion of CRPC Cells

**FIGURE 2:**
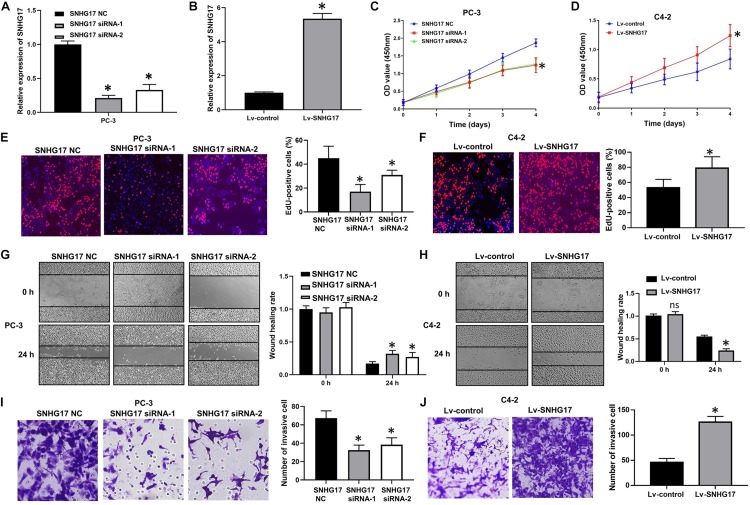
Effects of SNHG17 on cell proliferation and invasion of PC-3 cells *in vitro*. **(A)** The transfection efficacy of SNHG17 siRNAs in PC-3 cells, as detected by RT-QPCR; **(B)** Transfection efficacy of Lv-SNHG17 in C4-2 cells, as confirmed by RT-QPCR; **(C)** Effects of SNHG17 knockdown on the proliferation of PC-3 cells, as detected by CCK-8 assay; **(D)** Effects of SNHG17 overexpression on the proliferation of C4-2 cells, as detected by CCK-8 assay; **(E)** Effects of SNHG17 knockdown on the proliferation of PC-3 cells, as detected by EdU assay; **(F)** Effects of SNHG17 overexpression on the proliferation of C4-2 cells, as detected by EdU assay; **(G)** Effects of SNHG17 knockdown on the migration of PC-3 cells, as detected by the wound healing assay; **(H)** Effects of SNHG17 overexpression on cell migration of C4-2 cells, as detected by wound healing assay; **(I)** Effects of SNHG17 knockdown on the invasion of PC-3 cells, as detected by transwell assay; **(J)** Effects of SNHG17 overexpression on cell invasion of C4-2 cells, as detected by transwell assay. Data are shown as the mean ± *SE*. *P < 0.05, ns, not significant. The representative results of three independent experiments are shown.

Next, we determined the biological roles of SNHG17 in CRPC cell proliferation and invasion. RT-QPCR results confirmed that transfection with SNHG17 small interfering RNA (siRNA) stably down-regulated SNHG17 expression in PC-3 cells (*P* < 0.05, Figure 2A), whereas infection with Lv-SNHG17 stably up-regulated SNHG17 expression in C4-2 cells (*P* < 0.05, Figure 2B). Then, we investigated the effects of SNHG17 on CRPC cell proliferation, migration and invasion. CCK-8 assay revealed that the proliferation ability of SNHG17 siRNA transfected PC-3 cells was lower than that of SNHG17 NC transfected cells (*P* < 0.05, Figure 2C), but the proliferation ability of Lv-SNHG17-infected C4-2 cells was higher than that of Lv-control-infected cells (*P* < 0.05, Figure 2D). EdU assay revealed that the percentage of EdU positive of PC-3 cells was lower in the SNHG17 siRNAs groups compared with that in the SNHG17 NC group (*P* < 0.05, Figure 2E), but the percentage of EdU-positive C4-2 cells was higher in the Lv-SNHG17 group than that in the Lv-control group (*P* < 0.05, Figure 2F). In addition, as shown by the wound healing assay, the wound healing rates at 24 h post transfection of PC-3 cells with SNHG17 siRNA were enhanced compared with those of cells transfected with SNHG17 NC (*P* < 0.05, Figure 2G). However, the wound healing rates at 24 h post infection of C4-2 cells with Lv-SNHG17 were decreased compared with those of cells infected with Lv-control (*P* < 0.05, Figure 2H). Furthermore, the transwell assay showed that the number of invasive PC-3 cells in the SNHG17 siRNA group were less than the cells in the SNHG17 NC group (*P* < 0.05, Figure 2I), whereas the number of invasive cells in C4-2 cells infected with Lv-SNHG17 was lesser than that in cells infected with Lv-control (*P* < 0.05, Figure 2J). These results indicate that SNHG17 is a potential oncogenic lncRNA in CRPC, and can promote CRPC cell proliferation and invasion *in vitro*.

### SNHG17 Promotes Tumor Growth and Metastasis in CRPC *in vivo*

**FIGURE 3:**
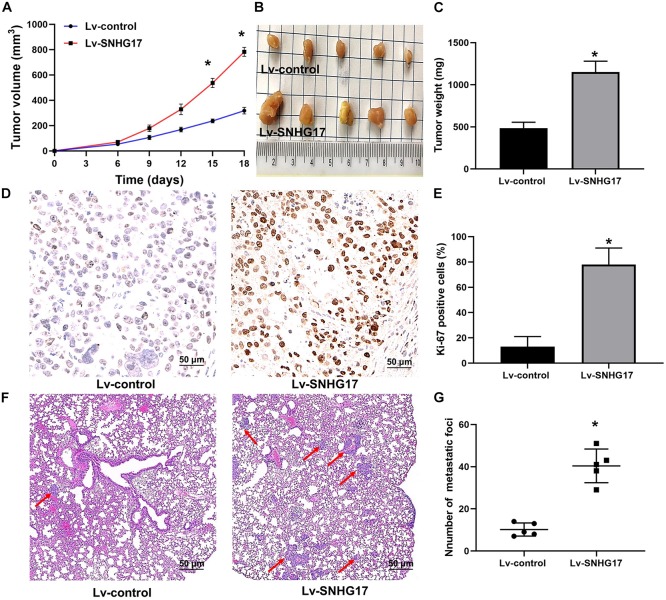
SNHG17 promotes tumor growth and metastasis in CRPC *in vivo*. **(A)** Tumor growth curve of C4-2 cells infected with Lv-control and Lv-SNHG17; **(B)** Digital images of all the resected tumors in cells transfected with Lv-control (*n* = 5) and Lv-SNHG17 (*n* = 5) were captured after 18 days; **(C)** Tumor weight of C4-2 cells infected with Lv-control and Lv-SNHG17; **(D)** Ki-67 expression in nude mice bearing C4-2 cells infected with Lv-control and Lv-SNHG17; **(E)** Statistical analysis of the percentage of Ki-67 positive cells in nude mice bearing C4-2 cells infected with Lv-control and Lv-SNHG17; **(F)** HE staining of metastatic foci in the lungs of nude mice injected with C4-2 cells transfected with Lv-control or Lv-SNHG17; **(G)** Statistical analysis of the total number of metastatic foci in 10 fields per lung section of Lv-control or Lv-SNHG17 group. Data are shown as the mean ± *S.E*. *P < 0.05. The representative results of three independent experiments are shown.

After C4-2 cells injection, faster tumor growth and larger tumor volumes were observed in the Lv-SNHG17 group (*P* < 0.05, Figures 3A,B). Consistently, tumor weight was found to be higher in the Lv-SNHG17 group than in the Lv-control group (*P* < 0.05, Figure 3C). Ki-67 staining showed that, compared to that in the Lv-control group, the number of Ki-67-positive cells was higher in the Lv-SNHG17 group (*P* < 0.05, Figures 3D,E). HE staining showed that the number of metastatic foci in the Lv-SNHG17 group was higher than in the Lv-control group in a pulmonary metastasis model of C4-2 cells (*P* < 0.05, Figures 3F,G). These results indicate that SNHG17 functions as an oncogenic lncRNAs to promote tumor growth and metastasis in CRPC *in vivo*.

### CD51 Is Up-Regulated by SNHG17 *in vitro* and *in vivo*

**FIGURE 4:**
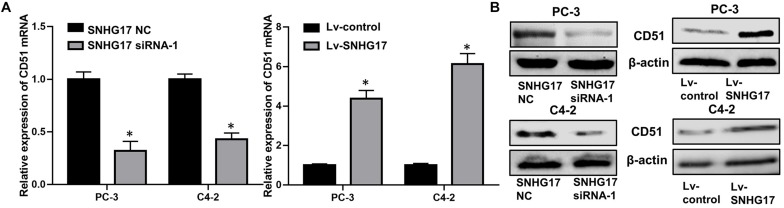
CD51 expression was up-regulated by SNHG17. **(A)** The effects of SNHG17 on the mRNA levels of CD51, as detected by RT-QPCR; **(B)** The effects of SNHG17 on the mRNA levels of CD51, as detected by WB; Data are expressed as the mean ± *S.E*. *P < 0.05, ns, not significant. The representative results of three independent experiments are shown.

RT-QPCR and WB analysis demonstrated that both the mRNA and protein levels of CD51 were down-regulated in CRPC cells transfected with SNHG17 siRNA compared to those in cells transfected with SNHG17 NC, but up-regulated in CRPC cells infected with Lv-SNHG17 compared to those in cells infected with Lv-control (*P* < 0.05, the effects on CD51 mRNA levels are shown in Figure 4A, the effects on CD51 protein levels are shown in Figure 4B). Furthermore, the expression of CD51 mRNA was up-regulated in cancer tissues of mice injected with Lv-SNHG17 infected CRPC cells (Supplementary Figures S1A,B). These results indicate that SNHG17 promotes CD51 expression *in vitro* and *in vivo*.

### CD51 Is the Functional Mediator of SNHG17

**FIGURE 5:**
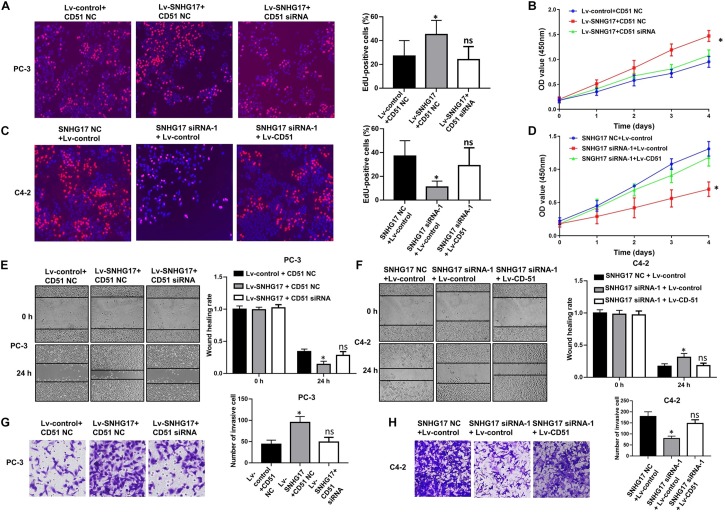
CD51 is involved in SNHG17-mediated CRPC cell proliferation and invasion. **(A)** The effects of down-regulation of CD51 on SNHG17 mediated cell proliferation of PC-3 cells, as detected by EdU assay; **(B)** The effects of down-regulation of CD51 on SNHG17 mediated cell proliferation of PC-3 cells, as detected by CCK-8 assay; **(C)** The effects of up-regulation of CD51 on SNHG17 mediated proliferation of C4-2 cells, as detected by EdU assay; **(D)** The effects of up-regulation of CD51 on SNHG17 mediated cell proliferation of C4-2 cells, as detected by CCK-8 assay; **(E)** The effects of down-regulation of CD51 on SNHG17 mediated migration of PC-3 cells, as detected by the wound healing assay; **(F)** The effects of up-regulation of CD51 on SNHG17 mediated migration of C4-2 cells, as detected by the wound healing assay; **(G)** The effects of down-regulation of CD51 on SNHG17 mediated cell invasion in PC-3 cells, as detected by the transwell assay; **(H)** The effects of up-regulation of CD51 on SNHG17 mediated cell invasion in C4-2 cells, as detected by the transwell assay; Data are expressed as the mean ± *S.E*. *P < 0.05, ns, not significant. The representative results of three independent experiments are shown.

In functional terms, EdU (Figure 5A), CCK-8 (Figure 5B), wound healing (Figure 5E), and transwell assay (Figure 5G) indicated that upregulation of SNHG17 significantly enhanced the proliferation, migration, and invasion of PC-3 cells (*P* < 0.05). Conversely, silencing of SNHG17 expression significantly decreased the proliferation (Figures 5C,D), migration (Figure 5F), and invasion (Figure 5H) of C4-2 cells (*P* < 0.05). Furthermore, the proliferation, migration and invasion of PC-3 cells were subsequently decreased after silencing of CD51 expression. Whereas the proliferation, migration and invasion of C4-2 cells were subsequently enhanced after upregulating CD51 expression (all *P* < 0.05, Figure 5). These results indicate that SNHG17 promotes CRPC cell proliferation, migration and invasion in CRPC, and silencing of CD51 expression ameliorates the effects of SNHG17 on CRPC cells.

### SNHG17 Directly Binds With miR-144 in CRPC Cells

**FIGURE 6:**
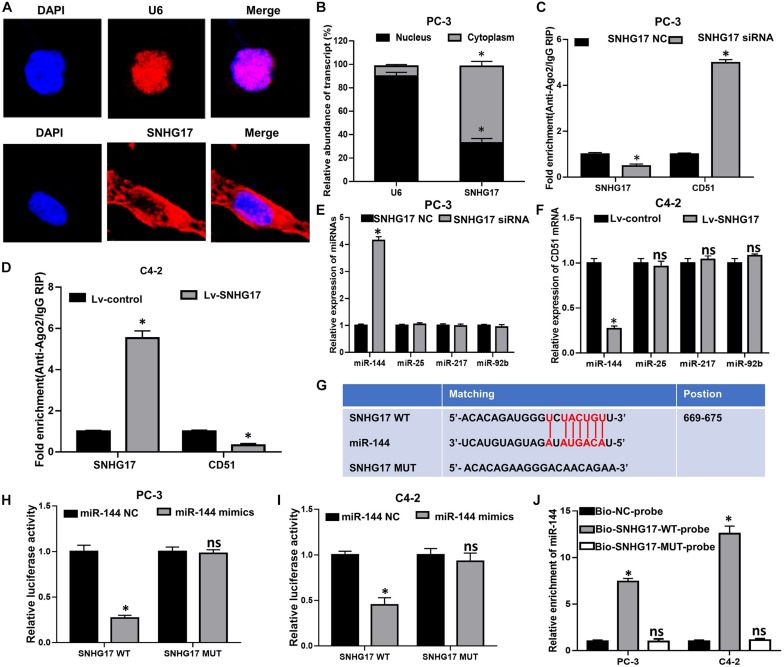
SNHG17 directly sponge miR-144 in CRPC cells. **(A)** Subcellular localization of SNHG17 in PC-3 cells, detected by FISH; Blue: DAPI, Red: CY3-SNHG17-probe. U6 was used as a nuclear marker; **(B)** Subcellular localization of SNHG17 in PC-3 cells, as detected by RT-QPCR. U6 was used as a nucleus marker; **(C,D)** Enrichment of SNHG17 and CD51 in Ago2 containing beads, detected in PC-3 **(D)** and C4-2 **(E)** cells by Ago2-RIP; **(E,F)** Effects of SNHG17 on the expression of miRNAs with putative binding sites for SNHG17 in PC-3 **(E)** and C4-2 **(F)** cells; **(G)** miR-144 WT and MUT targeting region of SNHG17; **(H,I)** Relative luciferase activity of PC-3 **(H)** and C4-2 **(I)** cells co-transfected with WT/MUT SNHG17 plasmid and miR-144 NC/mimics; **(J)** Enrichment of miR-144 in the sample pulled down by Bio-SNHG17-probe, Bio-SNHG17-MUT-probe, and Bio-NC-probe; Data are expressed as the mean ± *S.E*. *P < 0.05, ns, not significant. The representative results of three independent experiments are shown.

To further elucidate the underlying mechanism of SNHG17 in CRPC, FISH was performed to identify the subcellular localization of SNHG17 in PC-3 cells. FISH assay showed that SNHG17 was localized as a punctate pattern in both the cytoplasm and nuclei, but predominantly in the cytoplasm (Figures 6A,B), indicating that SNHG17 was more likely to function as a ceRNA in CRPC cells. Subsequently, we further explored whether SNHG17 could function as a ceRNA to enhance CD51 expression, and promote CRPC cell proliferation and invasion. We performed anti-Ago2 RIP in PC-3 cells transfected with SNHG17 siRNA and C4-2 cells infected with Lv-SNHG17 to investigate the enrichments of targets bound by miRNAs. Endogenous SNHG17 pull-down by Ago2 was significantly down-regulated in SNHG17 siRNA-transfected PC-3 cells (*P* < 0.05. Figure 6C), but significantly up-regulated in Lv-SNHG17-infected C4-2 cells (*P* < 0.05. Figure 6D). Conversely, endogenous CD51 pull-down by Ago2 was significantly up-regulated in SNHG17 siRNA-transfected PC-3 cells (*P* < 0.05, Figure 6C), but significantly down-regulated in Lv-SNHG17-infected C4-2 cells (*P* < 0.05, Figure 6D), indicating that there were less miRNAs bound CD51 transcripts in SNHG17-overexpressing cells and more miRNAs-bound CD51 transcripts in SNHG17 knockdown cells. Next, we predicted the potential target miRNAs of SNHG17 using miRNAda^2^ and starBase V2.0^3^. miR-25, miR-144, miR-217, and miR-92b showed the sequence complementarity with SNHG17 in both the miRNAda and starBase V2.0 databases. However, only miR-144 expression was found to be significantly up-regulated in SNHG17 knockdown PC-3 cells (*P* < 0.05, Figure 6E), and significantly down-regulated in SNHG17 overexpressing C4-2 cells (*P* < 0.05, Figure 6F). The expression levels of miR-137, miR-374, and miR-511 were not significantly altered following changes in SNHG17 expression in CRPC cells (*P* > 0.05, Figures 6E,F). More importantly, the expression of miR-144 was also down-regulated in cancer tissues of mice injected with Lv-SNHG17 infected CRPC cells (Supplementary Figure S1C). Thus, we constructed an SNHG17 luciferase reporter construct containing WT or MUT binding sites for miR-144 to further identify the association between SNHG17 and miR-144 in CRPC cells (Figure 6G). Results revealed that co-transfection of CRPC cells with SNHG17 WT vector and miR-144 mimics resulted in a significant decline in relative luciferase activity (*P* < 0.05, Figures 6H,I). However, the relative luciferase activities in CRPC cells co-transfected with SNHG17 MUT vector and miR-144 mimics were not significantly altered (*P* > 0.05, Figures 6G–I). More importantly, RNA pull-down assay demonstrated that miR-144 could be pulled down by Bio-SNHG17-probe, but not Bio-SNHG17-MUT-probe in CRPC cells (*P* > 0.05, Figure 6J). These results indicate that SNHG17 can directly sponge miR-144 in CRPC cells.

### miR-144 Inhibits the Proliferation and Colony Forming Ability of CRPC Cells

**FIGURE 7:**
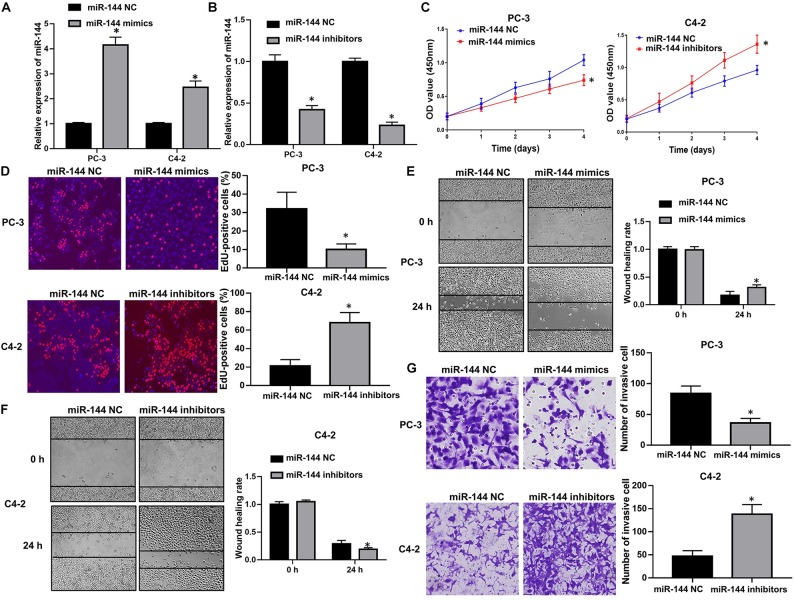
miR-144 inhibited CRPC cell proliferation and cell invasion. **(A)** Transfection efficacy of miR-144 mimics in CRPC cells, as detected by RT-QPCR; **(B)** Transfection efficacy of miR-144 inhibitors in CRPC cells, as detected by RT-QPCR; **(C)** The effects of miR-144 mimics or inhibitors on the proliferation of PC-3 and C4-2 cells, as detected by CCK-8 assay; **(D)** The effects of miR-144 on the proliferation of PC-3 and C4-2 cells, as detected by EdU assay; **(E)** The effects of miR-144 mimics on the migration of PC-3 cells, as detected by wound healing assay; **(F)** The effects of miR-144 inhibitors on the migration of C4-2 cells, as detected by wound healing assay; **(G)** The effects of miR-144 mimics or inhibitors on the invasion of PC-3 and C4-2 cells, as detected by transwell assay; Data are expressed as the mean ± *SE*. *P < 0.05, ns, not significant. The representative results of three independent experiments are shown.

We further explored the biological roles of miR-144 in CRPC cell proliferation and invasion. RT-QPCR results confirmed that transfection with miR-144 mimics stably up-regulated miR-144 expression in CRPC cells (*P* < 0.05, Figure 7A), whereas transfection with miR-144 inhibitors stably down-regulated miR-144 expression in CRPC cells (*P* < 0.05, Figure 7B). Next, we investigated the effects of miR-144 on CRPC cell proliferation, migration and invasion. CCK-8 assay showed that the proliferation ability of PC-3 cells transfected with miR-144 mimics was lower than that of cells transfected with miR-144 NC (*P* < 0.05, left panel, Figure 7C). Whereas the proliferation ability of C4-2 cells transfected with miR-144 inhibitors was higher than that of cells transfected with miR-144 NC (*P* < 0.05, right panel, Figure 7C). EdU assay revealed that the proportion of EdU-positive cells was lower in PC-3 cells transfected with miR-144 mimics than those in cells transfected with miR-144 NC (*P* < 0.05, upper panel, Figure 7D), but, higher in C4-2 cells infected with miR-144 inhibitors than those in cells infected with miR-144 NC (*P* < 0.05, lower panel, Figure 7D). In addition, as shown by the wound healing assay, the wound healing rates at 24 h post transfection of PC-3 cells with miR-144 mimics were enhanced compared with those in cells transfected with miR-144 NC (*P* < 0.05, Figure 7E). However, the wound healing rates at 24 h post infection of C4-2 cells with miR-144 inhibitors were decreased compared with those in cells infected with miR-144 NC (*P* < 0.05, Figure 7F). Furthermore, transwell assay showed that the number of invasive cells in PC-3 cells transfected with miR-144 mimics was less than those in cells transfected with miR-144 NC (*P* < 0.05, upper panel, Figure 7G). Whereas the number of invasive cells in C4-2 cells infected with miR-144 inhibitors was less than those in C4-2 cells infected with miR-144 NC (*P* < 0.05, lower panel, Figure 7G). These results indicate that miR-144 is a potential tumor suppressor in CRPC, and can inhibit CRPC cell proliferation and invasion.

### CD51 Is the Downstream Target of miR-144 in CRPC

**FIGURE 8:**
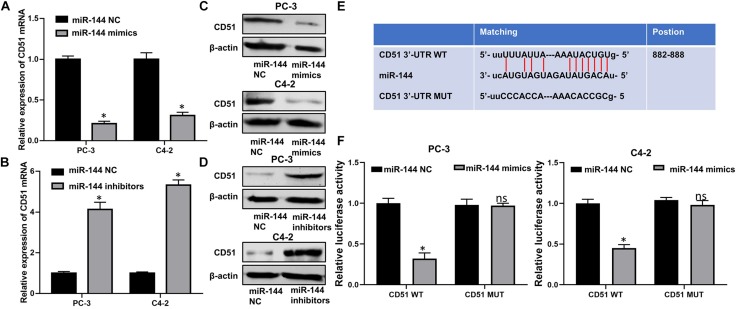
CD51 is a direct target of miR-144 in CRPC cells. **(A)** Effects of miR-144 mimics on the mRNA levels of CD51 in PC-3 and C4-2 cells, as detected by RT-QPCR; **(B)** Effects of miR-144 inhibitors on the mRNA levels of CD51 in PC-3 and C4-2 cells, as detected by RT-QPCR; **(C)** Effects of miR-144 mimics on the protein levels of CD51 in PC-3 and C4-2 cells, as detected by WB; **(D)** Effects of miR-144 inhibitors on the protein levels of CD51 in PC-3 and C4-2 cells, as detected by WB; **(E)** Putative binding sequence of miR-144 in 3’-UTR of CD51; **(F)** Relative luciferase activity of PC-3 and C4-2 cells co-transfected with WT/MUT CD51 plasmid and miR-144 NC/mimics; Data are expressed as the mean ± *S.E*. *P < 0.05, ns, not significant. The representative results of three independent experiments are shown.

Next, we investigated the correlation between miR-144 and CD51 expression in prostate cancer. As indicated by RT-QPCR and WB analyses, the mRNA and protein levels of CD51 were down-regulated in CRPC cells transfected with miR-144 mimics, but up-regulated in cells transfected with miR-144 inhibitor as compared to those in cells transfected with miR-144 NC (all *P* < 0.05, Figures 8A–D). Thus, we constructed a CD51 3′-UTR luciferase reporter containing WT or MUT binding sites for miR-144 to further identify the association between CD51 and miR-144 in CRPC cells (Figure 8E). Luciferase reporter assay revealed that co-transfection of CRPC cells with CD51 3′-UTR WT vector and miR-144 mimics resulted in a significant decline in relative luciferase activity (all *P* < 0.05) in PC-3 (left panel of Figure 8F) and C4-2 (right panel of Figure 8F) cells. However, the relative luciferase activity of CRPC cells co-transfected with CD51 3’-UTR MUT vector and miR-144 mimics was not significantly altered (*P* > 0.05, Figure 8). These results indicate that CD51 is a direct target of miR-144 in CRPC cells.

### CD51 Reverses the Biological Effects of miR-144 on CRPC Cells

**FIGURE 9:**
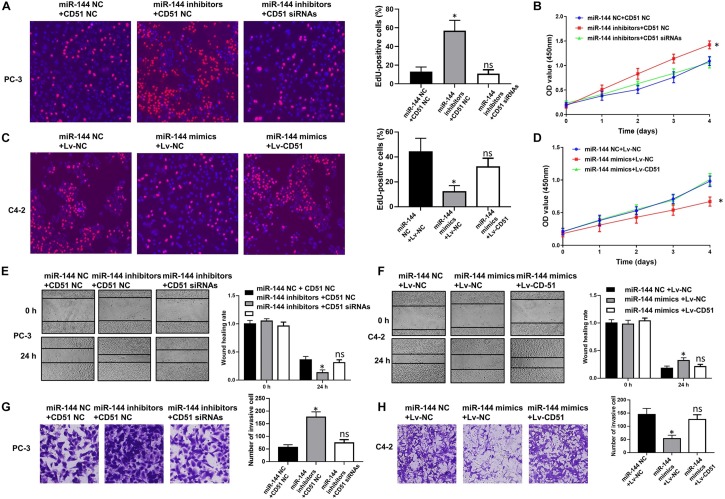
CD51 is the functional mediator of miR-144-induced proliferation and invasion of CRPC cells. **(A,C)** The effects of CD51 on miR-144 mediated proliferation of PC-3 **(A)** and C4-2 **(C)** cells, as detected by EdU assay; **(B,D)** The effects of CD51 on miR-144 mediated cell proliferation of PC-3 **(B)** and C4-2 **(D)** cells, as detected by CCK-8 assay; **(E)** The effects of CD51 siRNA on miR-144 inhibitors mediated migration of PC-3 cells; **(F)** The effects of Lv-CD51 on miR-144 mimics mediated migration of C4-2 cells; **(G,H)** The effects of CD51 on miR-144 mediated invasion of PC-3 **(G)** and C4-2 **(H)** cells, as detected by EdU assay; Data are expressed as the mean ± *SE*. *P < 0.05, ns, not significant. The representative results of three independent experiments are shown.

In functional terms, EdU (Figure 9A), CCK-8 (Figure 9B), wound healing (Figure 9E), and transwell assay (Figure 9G) indicated that downregulation of miR-144 significantly enhanced the proliferation, migration, and invasion of PC-3 cells (*P* < 0.05). Conversely, up-regulation of miR-144 significantly decreased the proliferation (Figures 9C,D), migration (Figure 9F), and invasion (Figure 9H) of C4-2 cells (*P* < 0.05). However, the proliferation, migration and invasion (Figure 9) of PC-3 cells were significantly decreased (*P* < 0.05) after silencing CD51 expression, but significantly increased after upregulating CD51 expression. These results indicate that miR-144 inhibits CRPC proliferation, migration and invasion, and CD51 ameliorates these miR-144-induced effects on CRPC cells.

## Discussion

LncRNAs have been identified as prognostic predictor and crucial regulator of multiple cancers, including prostate cancer (Ramnarine et al., 2019). Previously, by generating comparative lncRNA profiles in the CRPC cell line C4-2 and the HSPC cell line LNCaP, we found that the expression of Linc00963 was up-regulated in CRPC cells compared to that in HSPC cells (Wang et al., 2014). Furthermore, we also found that silencing Linc00963 expression in C4-2 cells attenuated cell proliferation, migration and invasion, and decreased EGFR and PIK3CA expression, and AKT phosphorylation. Interestingly, we also found that the expression of lncRNA VIM-AS1, PlncRNA-1, and SNHG17 were up-regulated in C4-2 cells compared to that in LNCaP cells (Wang et al., 2014), indicating that these lncRNAs might be involved in the transition from HSPC to CRPC. In another study, PlncRNA-1 was found to enhance cell proliferation and induce EMT in prostate cancer through reciprocal regulation of AR (Cui et al., 2013; Fang et al., 2016), Her-2 (Yang et al., 2017), and TGF-β (Jin et al., 2017) signaling pathways. SNHG17 has been identified as an oncogenic lncRNAs. Previous studies confirmed that SNHG17 promoted cell proliferation and migration by regulating FOXA1, XF1 and BIK expression in non-small cell lung cancer (Xu et al., 2019). Moreover, SNHG17 was identified as an unfavorable prognostic predictor in gastric cancer (Chen et al., 2019) and colorectal cancer (Ma et al., 2017). However, the functions and underlying mechanism of SNHG17 in the regulation of CRPC proliferation have not yet been elucidated. In the current study, we further confirmed the enhanced expression and oncogenic roles of SNHG17 in CRPC. Consistent with the findings of our previous study, we found that SNHG17 was up-regulated in CRPC tissues, and up-regulation of SNHG17 enhanced the proliferation, migration and invasion of CRPC cells (PC-3 and C4-2) *in vitro* and *in vivo*. Both our previous and current studies indicate that SNHG17 is up-regulated in CRPC, and is a potential oncogene in CRPC cells.

Mechanically, lncRNAs can bind with protein or miRNAs depending on their subcellular location. If lncRNAs are localized in the nucleus, they usually bind with protein to regulate target gene expression at the transcriptional levels. For example, lncRNA LBCS suppressed the castration resistance and proliferation of prostate cancer cells by functioning as a scaffold of hnRNPK protein and AR mRNA to inhibit AR translation efficiency (Gu et al., 2019). LncRNA HOXD-AS1 promoted the proliferation and chemo-resistance of CRPC cells by binding with WDR5 protein to mediate H3K4me3 modification in target genes (Gu et al., 2017). However, if lncRNAs are distributed mainly in the cytoplasm, they are likely to enhance target gene expression at the post-transcriptional level by acting as a ceRNA. For example, lncRNA FOXP4-AS1 promoted tumor growth and metastasis by targeting the miR-3284-5p/FOXP4 axis in prostate cancer (Wu et al., 2019). Moreover, lncRNA activated in prostate cancer progression (lncAPP) accelerated prostate cancer progression by competitively sponging miR-218 to enhance ZEB2 and CDH2 expression (Shi X. et al., 2019). A previous study showed that SNHG17 was located in the nucleus and could suppress P57 expression by binding with enhancer of zester homolog 2 (EZH2) in colorectal cancer (Ma et al., 2017) and gastric cancer (Zhang G et al., 2019). However, inconsistent with these previous studies, our findings revealed that SNHG17 was mainly located in the cytoplasm in CRPC cells, indicating that SNHG17 functioned as a ceRNA to regulate CRPC progression. The potential reasons that account for this discrepancy are the highly heterogeneous gene expression patterns and functions of lncRNAs with high tissue- and tumor-specificity. More studies are needed to further clarify the location and functions of SNHG17 in other types of cancers.

Numerous evidence indicates that up-regulation of CD51 is significantly associated with cancer progression and poor prognosis in multiple types of cancers, such as gastric cancer (Wang et al., 2019), osteosarcoma (Pei et al., 2019), colorectal cancer (Flum et al., 2018), and nasopharyngeal carcinoma (Ding et al., 2017). The expressions and functions of CD51 have been shown to be dysregulated in prostate cancer. Further, CD51 was identified as an important contributor to adhesion, EMT and tumor metastasis in prostate cancer (van der Horst et al., 2011; Ciardiello et al., 2019). Previously, we found that silencing CD51 expression would result in the loss of abilities of tumor initiation, migration, invasion, and chemoresistance in CRPC cells. Furthermore, CD51 is not only involved in the maintenance of stemness of pCSCs, but is also a marker of pCSCs to distinguish it from tumor cells (Sui et al., 2018). The oncogenic functions of CD51 in the carcinogenesis and progression of prostate cancer have been identified, and the relationships between CD51 and pCSCs has been preliminarily clarified by previous studies and our study. However, the regulatory mechanism of CD51 in CRPC has not been fully elucidated. In the current study, we preliminarily explore the mechanism by which CD51 exerts its oncogenic functions in CRPC. We found that CD51 expression was positively correlated with SNHG17 expression in CRPC tissues. Moreover, silencing of SNHG17 expression resulted in a significant decrease of CD51 expression in CRPC cells. Besides, we also found that silencing of CD51 expression ameliorated the oncogenic effects of SNHG17 on CRPC cell proliferation and invasion, indicating CD51, a known pCSC biomarker, is the down-stream target and functional mediator of SNHG17 in CRPC cells. More importantly, we also found that CD51 was the direct target of miR-144 in CRPC cells, and SNHG17 could directly bind with miR-144, thus indicating that the ceRNA regulatory mechanism might account for the regulation of CD51 expression in CRPC.

miR-144 can function as a tumor suppressor or oncogene depending on the type of cancer. For example, miR-144 has been identified as a tumor suppressive miRNA in multiple types of cancers, such as gastric cancer (Mushtaq et al., 2019), lung cancer (Jiang et al., 2019), cervical cancer (Shi F. et al., 2019), and colorectal cancer (Sheng et al., 2019). Specifically, miR-144 was down-regulated in colorectal cancer cells and tissues, and suppressed cancer development by directly targeting SMAD4 (Sheng et al., 2019) and CXCL11 (Han et al., 2018). Furthermore, miR-144 expression was lower in primary human osteosarcoma tissue samples and osteosarcoma cell lines (Zhao et al., 2014). Besides, up-regulation of miR-144 suppressed tumor growth and metastasis of osteosarcoma *in vivo* and *in vitro* by directly inhibiting RhoA and ROCK1 expression (Liu et al., 2019). However, miR-144 plays an oncogenic role in nasopharyngeal carcinoma and clear cell renal cell carcinoma. In these cancers, some established tumor suppressive genes were found to be the direct targets of miR-144, such as PTEN in nasopharyngeal carcinoma (Song et al., 2019), and ARID1A in clear cell renal cell carcinoma (Xiao et al., 2017). In prostate cancer, miR-144 was shown to enhance radiotherapy sensitivity and suppress hypoxia-induced autophagy by inhibiting PIM1 expression (Gu et al., 2016). Furthermore, miR-144 could decrease the proliferative ability and induce apoptosis in CRPC cells by targeting CEP55 (You and Zhang, 2018). Consistent with these findings, our results also confirmed miR-144 functioned as a tumor suppressive gene in CRPC. We provided evidence that overexpression of miR-144 significantly decreased CRPC cells proliferation, migration, and invasion. Functionally, CD51 was identified as the direct target and functional mediator of miR-144 in CRPC cells. Rescue experiments further confirmed that knockdown of CD51 reversed the effects of miR-144 inhibitors on cell proliferation and invasion. Hence, these results indicate that miR-144 inhibits CRPC proliferation by directly targeting CD51.

## Conclusion

We revealed that CD51 was the downstream target of both SNHG17 and miR-144. Moreover, CD51 expression was up-regulated by SNHG17 and down-regulated by miR-144 in CRPC cells. In the last, we elucidated SNHG17-facilitated CD51 expression and promoted cell proliferation and invasion of CRPC cells by sequestering miR-144. Thus, our study contributes to revealing the regulatory mechanism of SNHG17 and CD51 in CRPC and may provide novel biomarker for diagnosis and treatment of CRPC in the future.

## Data Availability Statement

All data generated in this study are available from the corresponding author on reasonable request.

## Ethics Statement

This study was reviewed and approved by the Ethnical Committee of The First Affiliated Hospital of Xi’an Jiaotong University. The protocol was performed in accordance with the Declaration of Helsinki and all participants enrolled in this study provided their written informed consent.

## Author Contributions

MB, YL, XM, MW, YD, and JM performed the research. SH designed the research study, PY contributed to essential reagents or tools. MB analyzed the data. SH wrote the manuscript. All authors have read and approved the final manuscript.

## Conflict of Interest

The authors declare that the research was conducted in the absence of any commercial or financial relationships that could be construed as a potential conflict of interest.

## Footnotes

1 https://www.ncbi.nlm.nih.gov/nuccore/NR_027241.3

2 http://www.microrna.org

3 http://starbase.sysu.edu.cn

## Supplementary Material

FIGURE S1 SNHG17 promotes CD51 and suppresses miR-144 expression in CRPC *in vivo*. **(A)** The effects of SNHG17 on the mRNA levels of CD51 *in vivo*, as detected by RT-QPCR. **(B)** The effects of SNHG17 on the protein levels of CD51 *in vivo*, as detected by immunofluorescence. **(C)** The effects of SNHG17 on the expression of miR-144 *in vivo*, as detected by RT-QPCR. Data are expressed as the mean ± SE. **P* < 0.05, ns: not significant. The representative results of 3 independent experiments are shown.
