# Supplementary figures and images for: Long Non-coding RNA SNHG17 Promotes Cell Proliferation and Invasion in Castration-Resistant Prostate Cancer by Targeting the miR-144/CD51 Axis
Source: Front Genet. 2020 Apr 15;11:274. doi: 10.3389/fgene.2020.00274 (PMC7174785; doi:10.3389/fgene.2020.00274)

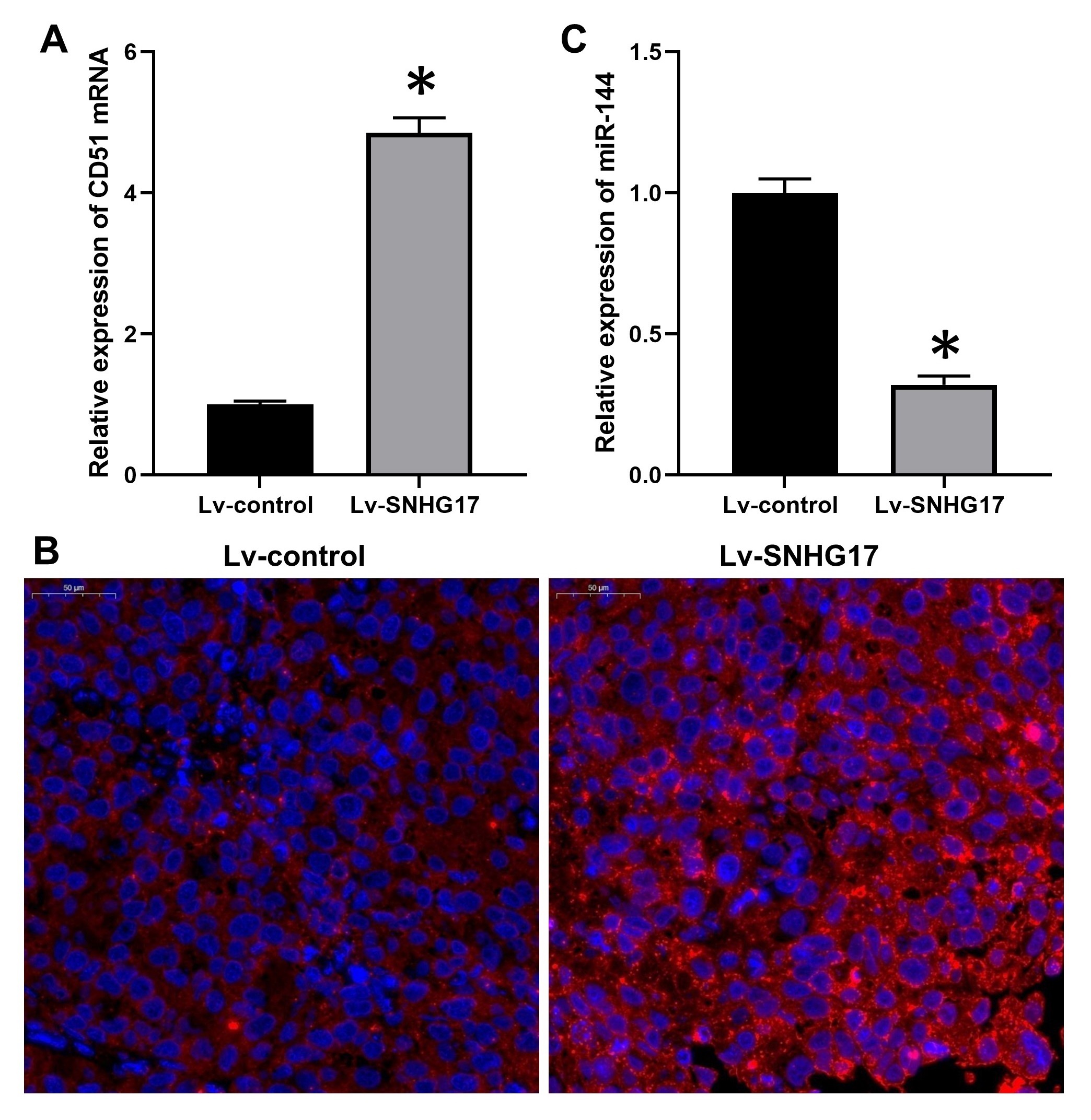

Supplement: FIGURE S1 — SNHG17 promotes CD51 and suppresses miR-144 expression in CRPC in vivo. (A) The effects of SNHG17 on the mRNA levels of CD51 in vivo, as detected by RT-QPCR. (B) The effects of SNHG17 on the protein levels of CD51 in vivo, as detected by immunofluorescence. (C) The effects of SNHG17 on the expression of miR-144 in vivo, as detected by RT-QPCR. Data are expressed as the mean ± SE. *P < 0.05, ns: not significant. The representative results of 3 independent experiments are shown. [file Image_1.JPEG]
